# Molecular pathogenesis of sporadic colorectal cancers

**DOI:** 10.1186/s40880-015-0066-y

**Published:** 2016-01-06

**Authors:** Hidetsugu Yamagishi, Hajime Kuroda, Yasuo Imai, Hideyuki Hiraishi

**Affiliations:** Department of Diagnostic Pathology, Dokkyo Medical University, 880 Kita-Kobayashi, Mibu, Shimotsuga, Tochigi 321-0293 Japan; Department of Pathology, International University of Health and Welfare Hospital, 537-3 Iguchi, Nasushiobara, Tochigi 329-2763 Japan; Department of Diagnostic Pathology, Ota Memorial Hospital, Fuji Heavy Industries Health Insurance Society, 455-1 Oshima, Ota, Gunma 373-8585 Japan; Department of Gastroenterology, Dokkyo Medical University, 880 Kita-Kobayashi, Mibu, Shimotsuga, Tochigi 321-0293 Japan

**Keywords:** Sporadic colorectal cancer, Pathogenesis, Morphology, Large-scale genome analysis, Genetic pathway

## Abstract

Colorectal cancer (CRC) results from the progressive accumulation of genetic and epigenetic alterations that lead to the transformation of normal colonic mucosa to adenocarcinoma. Approximately 75% of CRCs are sporadic and occur in people without genetic predisposition or family history of CRC. During the past two decades, sporadic CRCs were classified into three major groups according to frequently altered/mutated genes. These genes have been identified by linkage analyses of cancer-prone families and by individual mutation analyses of candidate genes selected on the basis of functional data. In the first half of this review, we describe the genetic pathways of sporadic CRCs and their clinicopathologic features. Recently, large-scale genome analyses have detected many infrequently mutated genes as well as a small number of frequently mutated genes. These infrequently mutated genes are likely described in a limited number of pathways. Gene-oriented models of CRC progression are being replaced by pathway-oriented models. In the second half of this review, we summarize the present knowledge of this research field and discuss its prospects.

## Introduction

Colorectal cancer (CRC) is the third most common cancer and the fourth leading cause of cancer-related death worldwide [1, 2]. In 2012, 1,360,600 new cases were diagnosed and 693,900 deaths were attributed to CRC [2]. CRCs occur sporadically in the majority of cases, and only 5%–10% are due to inherited mutations in well-known cancer-related genes. However, up to 25% of patients have a family history of CRC, suggesting a specific contribution by genes that have yet to be identified [3]. CRCs develop from normal colonic mucosa via progressive accumulation of genetic alterations, such as mutations in adenomatous polyposis coli (*APC*) during earlier stages and mutations in rat sarcoma viral oncogene homolog (*RAS*) and tumor protein 53 (*TP53*) during later stages [4]. However, one study reported that mutations of all three genes were found in only 7% of CRCs, suggesting that other genes may be involved in the tumorigenic process [5]. Genomic instability is a fundamental process in colorectal carcinogenesis [6], as demonstrated in a number of inherited CRCs, such as hereditary non-polyposis colon cancer/Lynch syndrome and Mut YH-associated polyposis [3, 7], which are caused by germ-line mutations of genes involved in DNA replication and repair. Chromosomal instability (CIN) is a process that generates gene deletions, duplications, and chromosomal rearrangements. CIN is the most common type of genomic instability, occurring in 70%–85% of CRCs, mainly in tumors proficient in DNA mismatch repair (MMR) [8]. Microsatellite instability (MSI) is caused by DNA MMR deficiency and is characterized by frequent mutations at simple nucleotide repeat sequences [9]. MSI accounts for approximately 15% of sporadic CRCs. CpG island methylator phenotype (CIMP) is also described as an epigenetic instability that influences CRC pathogenesis [10–12]. One type of genomic instability usually predominates in the development of a specific CRC, although MSI and CIMP often coexist [13]. In the first half of this review, we describe the genetic pathways of sporadic CRCs and their clinicopathologic features.

Recently, large-scale genome analyses have detected many infrequently mutated genes, as well as a small number of frequently mutated genes. Although the data obtained are enormous and complex, many infrequently mutated genes are likely described in a limited number of pathways. Gene-oriented models of CRC progression are being replaced by pathway-oriented models. In the second half of this review, we summarize the present molecular genetics of sporadic CRCs clarified by the recent advances of genome analytical techniques.

## Three major genetic pathways for sporadic CRCs

Sporadic CRCs occur in patients who have a median age of 70–75 years, and approximately 70% of CRCs develop in the distal colon. Many differences in clinicopathologic features exist between the proximal and distal colons. Genetically, sporadic CRCs develop by the accumulation of a series of abnormalities in tumor suppressor genes and oncogenes. Several investigators have postulated the adenoma-carcinoma sequence theory, in which *APC* mutation serves as an initiating event, followed by the accumulation of multiple mutations of genes, such as Kirsten RAS (*KRAS*), *Sma*- and *Mad*-*related protein 4* (*SMAD4*), and *TP53* [4, 14, 15]. According to this model, at least seven distinct mutations are required for CRC pathogenesis. Other investigators have described another route to colorectal carcinogenesis through serrated polyps [16].

Presently, three major distinct genetic pathways to CRC have been postulated. Approximately 70% of sporadic CRCs develop along the CIN pathway. These cancers are characterized by the accumulation of numerical or structural chromosomal abnormalities, resulting in aneuploid karyotype, frequent loss-of-heterozygosity (LOH) at tumor suppressor gene loci, and chromosomal rearrangements [17]. Moreover, CIN tumors are distinguished by the accumulation of mutations in specific oncogenes and tumor suppressor genes [e.g., *APC*, *KRAS*, phosphatidylinositol-4,5-bisphosphate 3-kinase, catalytic subunit alpha (*PIK3CA*), B-Raf proto-oncogene, serine/threonine kinase (*BRAF*), *SMAD4*, and *TP53*], thereby activating pathways critical for carcinogenesis.

Another important pathway is the MSI pathway, caused by dysfunction of DNA MMR genes. MSI is found in 15% of sporadic CRCs. Unlike Lynch syndrome that is caused by germ-line mutations of MMR genes, such as MutL homolog 1 (*MLH1*) (32% of cases), MutS homolog 2 (*MSH2*) (39%), postmeiotic segregation increased 2 (*PMS2*) (15%), and *MSH6* (14%) [18], MMR deficiency in sporadic CRCs is due mainly to silencing of the MMR genes, mostly *MLH1* (>80% of cases), by promoter hypermethylation [19, 20]. Usually, expression is lost in the case of MLH1 and MSH2 and their binding partners (MSH6 and PMS2, respectively). Classification of MSI is based on altered size of various mono- and di-nucleotide repeat sequences, such as BAT25, BAT26, D2S123, D5S346, and D17S250, known as the Bethesda panel [21, 22]. Altered size of at least two of the five microsatellite panel markers is defined as MSI-high (MSI-H). Sporadic MSI-H is associated with CIMP. Most MSI-H CRCs are diploid or near diploid, and LOH is rare. CRCs with one abnormal marker in the panel are termed MSI-low (MSI-L), and their clinical significance is controversial. MSI-L is often grouped with microsatellite-stable (MSS) tumors. MSI-H tumors frequently have frameshift mutations in those genes that contain small runs of nucleotide repeats in exon-coding regions, such as transforming growth factor-β receptor 2 (*TGFBR2*), insulin-like growth factor 2 receptor (*IGF2R*), E2F transcription factor 4, p107/p130-binding (*E2F4*), *MSH6*, *MSH3*, and caspase 5 (*CASP5*) [23–28]. An (A)_10_ repeat of the *TGFBR2* gene is mutated in 80% of MSI-H CRCs. In MSI-H tumors, *APC* and *BRAF* are often mutated, but *KRAS* mutation is rare. Sporadic MSI-H CRCs, as well as those with Lynch syndrome, are characterized by right-sided location, mucinous or medullary type, and presence of tumor-infiltrating lymphocytes, earlier stages, and better prognoses [29, 30].

The third pathway, designated as CIMP, is characterized by a widespread CpG island methylation [10]. Approximately 30%–40% of sporadic proximal CRCs are CIMP-positive, compared with 3%–12% of distal CRCs [31–33]. CIMP-positive CRCs often have MSI-H due to methylation of the *MLH1* promoter, but more than 50% of CIMP tumors are MSS. CIMP is uncommon in Lynch syndrome that exhibits MSI [11, 34]. CIMP is also associated with *BRAF* mutations in both MSI and MSS CRCs [11, 35]. No consensus exists yet for what constitutes the optimal panel of CpG sites for CIMP determination. The classic panel consists of CpG sites in *MLH1*, cyclin-dependent kinase inhibitor 2A (*CDKN2A*, *p16*), and methylated in tumors (*MINTS*) 1, 2, and 31 [36]. CIMP-positive tumors based on the classic panel can be divided in two types, namely CIMP-high, related to *BRAF* mutations and *MLH1* methylation, and CIMP-low, related to *KRAS* mutations and MSS [37]. CIMP-negative tumors are MSS with frequent *TP53* mutation [37, 38]. Based on a systematic screen of 195 CpG sites, calcium channel, voltage-dependent, T type, alpha 1G subunit (*CACNA1G*), *IGF2*, neurogenin 1 (*NEUROG1*), runt-related transcription factor 3 (*RUNX3*), and suppressor of cytokine signaling 1 (*SOCS1*) was proposed as an alternative to the classic panel [20]. CIMP, which was defined by this panel, did not show a relationship to *KRAS*, but did strongly associate with *BRAF* V600E mutation [34, 39].

The definition of the three genetic pathways is not mutually exclusive, as in the case of CIMP, which often results in *MLH1* promoter methylation and MSI. Up to 25% of MSI CRCs can exhibit CIN [8], and concomitant CIMP and CIN were noted in 68 of 364 CRCs with CIN and 95 CRCs with CIMP [40].

## Genetic pathways and morphology

Consistent with genetic models, there appear to be at least three distinct clinicopathologic evolutional routes to sporadic CRCs [41, 42]. The first is the traditional pathway, which starts from normal mucosa via tubular adenomas (with *APC* mutations) and results in typical CRC in the distal colon (with *TP53* mutation and CIN). The second is the serrated pathway, which starts from normal mucosa via serrated adenomas (with *BRAF* mutations and CIMP) and results in colon cancer in the proximal colon with good prognosis (with *MLH1* loss and MSI). The third is the alternative pathway, which starts from normal mucosa via villous, partly serrated adenomas (with *KRAS*, *BRAF*, and *APC* mutations and CIMP) and results in colon cancer with poor prognosis (with CIMP). The traditional and serrated pathways are homogenous, but the alternative pathway is more heterogeneous. The prevalence of each pathway is estimated at 50%–70% (traditional), 10%–20% (serrated), and 10%–30% (alternative) [42]. In addition, superficial-type colorectal tumors and de novo cancer without precursor lesions have been identified. *KRAS* mutation was rare in superficial-type adenoma and adenocarcinoma [43, 44]. *TP53* and *APC* were frequently mutated, but *KRAS* was not mutated in de novo cancers, which were also significantly associated with LOH at chromosome 3p [45, 46].

**Fig. 1:**
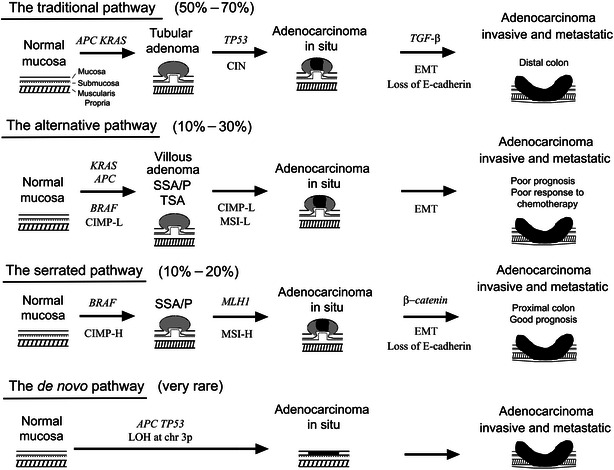
Evolutional pathways for colorectal morphogenesis. The traditional pathway is the most homogenous pathway, originating from tubular adenoma (via adenomatous polyposis coli (*APC*) and subsequently Kirsten rat sarcoma viral oncogene homolog (*KRAS*) mutation) and leading to adenocarcinoma [via tumor protein 53 (*TP53*) mutation]. This pathway is characterized by chromosomal instability (CIN), negative CpG island methylator phenotype (CIMP), and average outcome. The serrated pathway is also the most homogenous pathway, originating from sessile serrated adenoma/polyps (SSA/P) via B-Raf proto-oncogene, serine/threonine kinase (*BRAF*) mutation and CIMP-high (CIMP-H) and leading to adenocarcinoma via MutL homolog 1 (*MLH1*) promoter methylation and microsatellite instability-high (MSI-H). This pathway is characterized by good prognosis. The alternative pathway is more heterogeneous and may arise mostly from villous adenoma and perhaps also from SSA/P and traditional serrated adenoma (TSA) via CIMP-low (CIMP-L) and predominant *KRAS* but occasional *BRAF* mutations. This pathway lacks CIN and has the worse prognosis with low responsiveness to chemotherapy. The de novo cancers usually lack *KRAS* mutation but are significantly associated with *TP53* and *APC* mutations and also loss of heterozygosity (LOH) at chromosome 3p (chr 3p). *EMT* epithelial-mesenchymal transition, *TGF*-*β* transforming growth factor-β, *MSI-L* MSI-low. A part of this figure was reproduced from figure 1 in Patholog Res Int 2012;2012:509348 authored by Pancione et al. [42], with permission

CRCs in the late stage acquire more aggressive phenotypes and are more invasive and metastatic, for which epithelial-mesenchymal transition (EMT) has been proposed as a critical step [42]. EMT represses cell adhesion molecules, such as E-cadherin and zona occludens 1, and induces mesenchymal markers, such as vimentin and N-cadherin. Consequently, cells acquire a fibroblast-like appearance. Some growth factors, such as transforming growth factor-β (TGF-β), appear to be responsible for the induction of EMT, and the wingless/int-1 (WNT)/β-catenin signaling pathway and loss of E-cadherin are considered the major effectors of EMT. These pathways are summarized in Fig. 1.

The origin of CRCs in the colonic crypt has been investigated in association with these pathways, whether they are identical or truly different in characteristics and differentiation status [42]. It has been suggested that tumor development through the traditional pathway is slow (5–20 years) and that the initial events occur in the fully differentiated cells of the colonic crypt [42]. *APC* mutations have been detected in the cells of the upper crypt compartment [47], and the precursor lesions tend to grow upward. In contrast, tumor development through the serrated pathway may occur in the cells of the lower crypt compartment, whose functions are finely regulated by epigenetic mechanisms [48–50]. The precursor lesions of the serrated pathway grow downward or laterally, are rapidly progressive, and are prone to CIMP [42, 51].

**Fig. 2:**
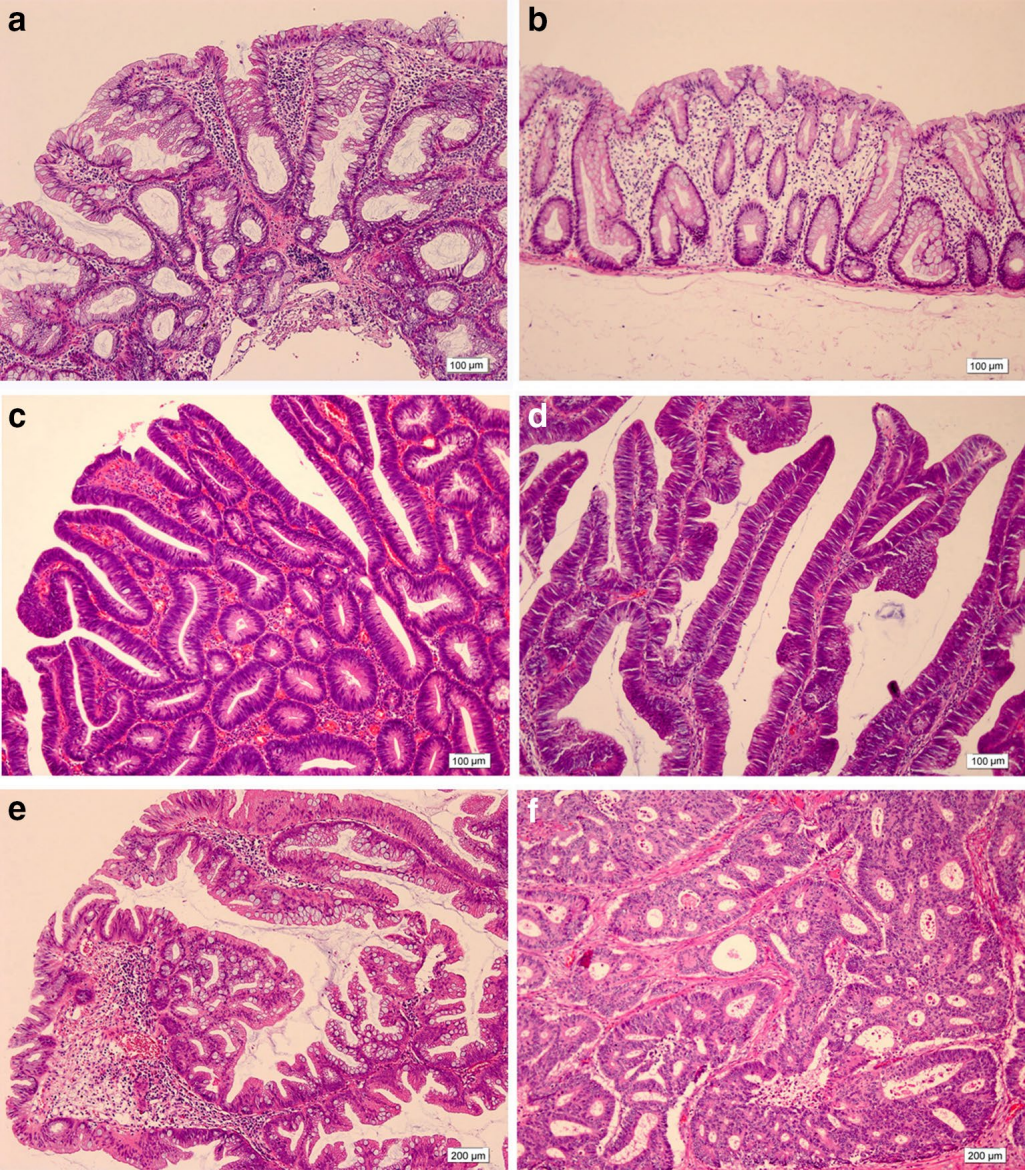
Representative histological figures of polyps, adenoma, and carcinoma of the colon and rectum (hematoxylin and eosin stain). **a** hyperplastic polyp. **b** sessile serrated adenoma/polyp. **c** tubular adenoma. **d** villous adenoma. **e** traditional serrated adenoma. **f** adenocarcinoma

Previously, hyperplastic polyps were considered non-neoplastic, but some are now considered precursors of CRC. Therefore, hyperplastic polyps were renamed “serrated polyps,” which the World Health Organization now classifies three categories: hyperplastic polyps, sessile serrated adenoma/polyps (SSA/Ps), and traditional serrated adenomas (TSAs) [16]. Hyperplastic polyps are most commonly located in the distal colon and typically exist as multiple sessile lesions of 1–5 mm in diameter. Histologically, the crypts are straight, and proliferation is located in the lower third of the crypts, with serration developing in the more luminal aspects [52]. SSA/Ps are more likely located in the proximal colon and are usually larger than hyperplastic polyps [53]. SSA/Ps, which comprise approximately 15%–20% of all serrated polyps, are thought to be precursors of sporadic CRC with MSI-H (serrated pathway) and probably with CIMP-positive MSS (alternative pathway). SSA/Ps show an overall distortion of crypts resulting from alterations of the proliferative zone, which is not located in the base of the crypt [52]. Crypts still reach the muscularis mucosa, and they are generally L-, inverted T-, or anchor-shaped. TSAs, which comprise less than 1% of all serrated polyps, may be precursors of sporadic CRC via the alternative pathway. They are usually located in the distal colon and characterized by an overall complex and villiform growth pattern, often with cells showing cytological dysplasia. Ectopic crypt formation, meaning crypts that do not reach the muscularis mucosa, is characteristic to TSA. TSAs show increased methylation but never show methylation of *MLH1*. Approximately 20%–50% of TSAs have *KRAS* mutation, and 30%–70% of TSAs have *BRAF* mutations [54–56]. *BRAF*-mutant and *KRAS*-mutant TSAs are thought to be disparate with distinct clinicopathologic and molecular features, although they cannot be distinguished morphologically. The *BRAF*-mutant TSAs, via *CDKN2A* silencing, represent a precursor of more aggressive MSS CRC [55]. Representative histologies of polyps, adenoma, and carcinoma of the colon and rectum are shown in Fig. 2.

## Large-scale genome analyses of sporadic CRCs

In 2007, Wood et al. [57] reported the results of exome-wide sequencing analyses of 11 CRCs. Most of the mutated genes (49–111; average, 76) per colorectal tumor were harmless or passengers, and fewer than 15 mutations were considered “driving mutations.” Non-synonymous mutations in CRCs showed a strong predilection for C to T transitions at the CpG dinucleotides.

In 2008, Leary et al. [58] reported the data of genome-wide copy number changes of 36 CRCs, homozygous deletion and amplification. Individual CRCs had on average seven copy number alterations, i.e., four homozygous deletions and three amplifications. The average number of protein-coding genes affected by homozygous deletion and amplification was nine per CRC. The data were integrated with those of the previous transcript mutational analysis [57], and candidate driver genes were identified: oncogenes, such as v-myc avian myelocytomatosis viral oncogene homolog (*MYC*), endothelial precursor protein B9 (*EPPB9*), epidermal growth factor receptor (*EGFR*), insulin receptor substrate 2 (*IRS2*), zinc finger protein 480 (*ZNF480*), *ZNF155*, and Neugrin (*NGRN*), and tumor suppressor genes, such as phosphatase and tensin homolog (*PTEN*), *TP53*, mitogen-activated protein kinase kinase 4 (*MAP2K4*), *SMAD2*, *SMAD3*, *ZNF521*, and OMA1. A statistical approach was performed to examine whether groups of genes belonging to certain cellular pathways were preferentially affected by genetic alterations, and many genetic alterations were found to be described in a limited number of pathways, such as the EGFR-phosphatidylinositol 3-kinase (PI3K), EGFR-mitogen-activated protein kinase (MAPK), Notch, G_1_/S cell cycle transition pathways, cell–cell interaction and adhesion, and proteolysis pathways. For example, the EGFR signaling pathway via phosphatidylinositol(3,4,5)-triphosphate (PIP3) consists of 23 genes, in which 9 were found to be altered: 12 point mutations were found, 5 were amplified, and 6 were homozygously deleted. The net effect of a pathway can be the same whether certain components are altered by point mutations, amplifications, and deletions. These data suggest that the gene-oriented models of cancer progression are being replaced by the pathway-oriented models.

In 2011, Bass et al. [59] reported the results of whole-genome sequencing of nine CRCs and paired non-neoplastic controls. They found 137,968 somatic mutations in nine MSS-CRCs, with an average of 15,330 mutations per tumor. Non-synonymous coding mutations were 47–147 per tumor, with an average of 79. In the nine CRCs, they also found 675 genomic rearrangements (range, 5–182; mean, 75), of which 82% were intra-chromosomal. They found 11 rearrangements (2 inter- and 9 intra-chromosomal) that gave rise to in-frame fusion transcripts. By screening 97 more primary CRCs, an intra-chromosomal fusion on chromosome 10, vesicle transport through interaction with t-SNAREs 1A (*VTI1A*)-transcription factor 7-like 2 (*TCF7L2*), was found to be recurrently expressed (3% of CRCs). *TCF7L2* encodes a transcription factor TCF4 that dimerizes with β-catenin [encoded by catenin beta 1 (*CTNNB1*)] [60]. Small interfering RNA-mediated knockdown of *VTI1A*-*TCF7L2* resulted in a reduction in the anchorage-independent growth of NCI-H508-derived cells, which expressed the *VTI1A*-*TCF7L2* fusion [59]. These data suggest that functionally important fusions may occur in a small proportion of this disease.

In 2012, the Cancer Genome Atlas Network [61] published the results of genome-scale analyses of 276 CRCs, including exome sequencing, DNA copy number, promoter methylation, and mRNA and microRNA expression. The whole-exome sequencing of 224 CRCs revealed that hypermutated CRC (>12 mutations/Mb) comprised 16% of total samples, of which three-quarters were expected as MSI-H, usually with *MLH1* methylation; the other quarter was neither MSI-H nor CIMP but showed somatic mutations in one or more MMR genes and polymerase ε. The eight most frequently mutated genes among the non-hypermutated tumors (<8.24 mutations/Mb) were *APC*, *TP53*, *KRAS*, *PIK3CA*, F-box and WD repeat domain containing 7 (*FBXW7*), *SMAD4*, *TCF7L2*, and *NRAS*. *CTNNB1*, *SMAD2*, family with sequence similarity 123B (*FAM123B*), and SRY-box 9 (*SOX9*) were also mutated frequently. The eight most frequently mutated genes among the hypermutated CRCs were *BRAF* (*V600E*), activin receptor type-2A (*ACVR2A*), *APC*, *TGFBR2*, *MSH3*, *MSH6*, solute carrier family 9, subfamily A (NHE9, cation proton antiporter 9), member 9 (*SLC9A9*), and *TCF7L2*. The different sequences of genetic events were suggested between the two CRCs.

The Cancer Genome Atlas Network researchers investigated somatic copy number alterations in 257 tumors and identified potential targets of arm-level changes, including gains of 1q, 7p, 7q, 8p, 8q, 12q, 13q, 19q, 20p, and 20q, and losses of 1p, 4q, 5q, 8p, 14q, 15q, 18p and q including *SMAD4* (in 66% of the tumors), 17p and q including *TP53* (in 56% of the tumors), 20p, and 22q. In addition, they identified 28 regions of significant focal deletions and 17 regions of significant focal amplifications. One of the most common focal amplifications, found in 7% of the tumors, was a 100 to 150-kb region in 11p15.5, which contained genes encoding *IGF2* and *miR*-*483*. *IGF2* amplification/overexpression was found to be exclusive to the events leading to the activation of the PI3K pathway (*IRS2* up-regulation, *PIK3CA* mutation, and *PTEN* homozygous deletion), suggesting the importance of the IGF2-IGF1R-IRS2 axis signals to PI3K.

In addition, they identified 250 inter-chromosomal translocations in 97 tumors by whole-genome sequencing. They found three cases (3%) of neuron navigator 2 (*NAV2*)-*TCF7L1* fusions and 21 cases (22%) of translocation involving tetratricopeptide repeat domain 28 (*TTC28*), which is predicted to inactivate TTC28. *TTC28* is a target of TP53, and TTC28 protein inhibits tumor cell growth [62]. However, Pitkänen et al. [63] later reported that translocations involving *TTC28* may be long interspersed element-1 (LINE-1) transpositions originating from a LINE-1 element in the first intron of *TTC28*, and that such changes would be passengers resulting from promoter-hypomethylation and genetic instability observed in CRCs.

The recurrent genomic alterations were grouped in five pathways: the WNT, receptor tyrosine kinase (RTK)-RAS (MAPK), PI3K, TGF-β, and TP53 pathways (Table 1). Considerable overlap existed in the alterations of these pathways. The WNT pathway was altered in 93% of all tumors, and co-occurrence of alterations involving the RAS and PI3K pathways were observed in one-third of tumors.

**Table 1:** Frequencies of genetic changes leading to deregulation of the signaling pathways in colorectal cancers

Pathway	Frequency of genetic changes leading to deregulation	
	Non-hypermutated (%)	Hypermutated (%)
WNT signaling	92	97
TGF-β signaling	27	87
PI3K signaling	50	53
RTK-RAS signaling	59	80
TP53 signaling	64	47

Reproduced from Fig. 4 in Nature 2012;487:330–7 authored by the Cancer Genome Atlas Network [61] with modifications, with permission

*WNT* wingless/int-1, *TGF-β* transforming growth factor β, *PI3K* phosphatidylinositol 3-kinase, *RTK* receptor tyrosine kinase, *RAS* rat sarcoma viral oncogene homolog, *TP53* tumor protein 53

## Future prospects

Starting from mutational analyses of candidate genes, recent advances in analyzing genomic structure are greatly increasing our knowledge of CRC. Many infrequent gene alterations are going to be found. The immense complexity of cancer genome is somewhat misleading because most alterations are immaterial to neoplasia and are simply passenger changes. However, some of the infrequent mutations can be drivers by functioning through a much more limited number of the same signal transduction pathways. At present, all of the known driver genes for carcinogenesis can be classified into 12 pathways, which can be further classified into three core cellular processes: cell fate determination, cell survival, and genome maintenance, through which a growth advantage can be developed [64]. Thus, the gene-oriented models of cancer progression are being replaced by the pathway-oriented models.

The genome-wide data hitherto reported were obtained by analyzing CRCs in the later stage. Even the initial events that determine the fate of tumor cells, whether hypermutated or non-hypermutated, have not been elucidated yet, nor has the subsequent accumulation process of gene mutations/genomic alterations been elucidated. To date, CRCs have been subclassified by pathologic investigation. Genetic changes that underlie specific histological subtypes are largely unknown. Various stages of lesions must be analyzed, from normal-appearing epithelium to late-stage cancers. It may also be necessary to analyze cells at specific sites of the crypt. In addition, little is known about genetic differences between cancers arising de novo and those arising via adenoma. The data of arm-level changes reported by the Cancer Genome Atlas Network did not detect loss of 3p that had been associated previously with de novo cancers [46, 61]. Further investigations will be needed to clarify the whole picture of molecular carcinogenesis of CRCs.

Cancer genome sequencing has already had an impact on the clinical care of cancer patients. The detection of activating mutations in driver genes encoding protein kinases has led to the development of small-molecule inhibitor drugs targeting those kinases. On the other hand, more than half of the driver genes encode tumor suppressors. Small-molecule drugs cannot generally replace functions of defective gene products resulting from mutations in tumor suppressor genes. However, every tumor suppressor gene inactivation is expected to result in the activation of some growth-promoting signal downstream of the pathway, such as *PTEN* mutation that results in the activation of AKT kinase. The whole-genome analyses in respective patients will identify many less frequent driver mutations, which will help identify potential drug targets in each patient, leading to more personalized cancer therapy.

## Conclusions

Molecular genetics of sporadic CRCs has significantly advanced over the past two decades. The field has entered a new era, when the gene-oriented models are being replaced by the pathway-oriented models. Whole-genome analyses will further provide a useful resource for understanding CRCs and identifying possible molecular targets for their therapies.
